# Tokyo Teen Cohort study: a prospective cohort study on general population of adolescents

**DOI:** 10.1192/j.eurpsy.2023.997

**Published:** 2023-07-19

**Authors:** S. Ando, A. Nishida, S. Yamasaki, K. Endo, M. Hiraiwa-Hasegawa, K. Kasai

**Affiliations:** 1 The University of Tokyo; 2 Tokyo Metropolitan Institute of Medical Science, Tokyo; 3 SOKENDAI, Kanagawa, Japan

## Abstract

**Introduction:**

Adolescence is the period when many mental disorders have their peaks of onsets. Investigation into adolescent mental health problems and their risk factors is required, but there has been few prospective cohort studies on adolescent mental health.

**Objectives:**

This study aimed to prospectively reveal the developmental trajectory of physical and mental health in adolescence, and to investigate factors associated with the trajectory.

**Methods:**

We launched a prospective cohort study (Tokyo Teen Cohort: TTC) on general population of adolescents at three municipalities in metropolitan area in Tokyo, Japan. Using the resident register, we recruited 10-year-old children from the community between 2012 and 2015. The second, third, and fourth wave of data collection were conducted at 12, 14, 16 years of age, respectively. We collected multidisciplinary data including mental health by self-report questionnaire and home-visit interview. Further, we have launched two subsample studies which focus on biological measures such as brain MRI, EEG, and sex hormones. TTC is based at three research institutes, and ethics approval has been granted by all of the three institutions.

**Results:**

A total of 3171 children participated the TTC. Of those, 3007 children participated in the second wave of data collection (follow-up rate: 94.8). The third and fourth wave of data collection were completed and more than 80% of children continued to participate in TTC. More than 300 children participated in the two subsample studies. More than 30 papers were already published, and many national/international research collaborations have started.

**Conclusions:**

The fifth wave of data collection at 20 years of age is being currently conducted. Further national/international collaborations are expected to examine cultural effects on mental health of adolescents.

**Disclosure of Interest:**

None Declared

